# The Verification Process of a POC Blood Gas Analyser—The Nova Stat Profile Primer Plus Analyser

**DOI:** 10.1002/jcla.70006

**Published:** 2025-03-25

**Authors:** Joseph Costa, Ritienne Attard, the GGH Medical Laboratory Services team

**Affiliations:** ^1^ GGH Medical Laboratory Services Gozo General Hospital Victoria Gozo Malta

**Keywords:** bias, blood gas analyser, comparison, linearity, precision, verification

## Abstract

**Background:**

Point‐of‐care testing of blood gases plays a critical role in patient management. The aim of this study was to verify the manufacturer's specifications of the Nova Stat Profile Prime Plus Analyser, along with a comparison study with the GEM Premier 4000 Blood Gas Analyser.

**Methods:**

Parameters analysed were pH, pCO_2_, pO_2_, Na^+^, Cl^−^, K^+^, iCa, lactate, and glucose. Data for the precision and bias study were generated using control samples in a 5 × 5 study design. Linearity was checked using a five‐level Linearity Control Set, while comparison was done between the Nova and GEM analysers using whole blood samples (*N* = 103). Acceptance was based on the CLIA TE_a_ for all analytes except for lactate, for which the TE_a_ defined by CAP and AAB was used.

**Results:**

The within‐run and between‐run CV_R_% precision were all lower than the claimed CVs%, except for pCO_2_ control level 2 within run (CV% 1.5 [claim CV% 1.1]) and iCa control level 5 between run (CV% 1.42 [claim 1.12]). The observed bias for all parameters was within the calculated lower and upper bias limits. Linearity was verified for all analytes except for Na^+^. Upon comparison of the Nova and GEM analysers, a correlation coefficient above 0.95 was observed for most parameters.

**Conclusion:**

The Nova Stat Profile Prime Plus analyser meets the manufacturer's precision and bias claims. Linearity was confirmed for most analytes. There was a good correlation between the Nova and GEM Blood Gas analyser at concentrations within the reference range intervals for all investigated parameters.

## Graphical abstract

Point‐of‐care (POC) testing of blood gases plays a critical role in the management of patients both in the emergency department and in a critical care setting. Findings from this study confirm that the Nova Stat Profile Prime Plus analyser meets the manufacturer's precision and bias claims. Linearity was confirmed for pCO2, pO2, K+, Cl‐ and iCa. A good correlation was found between the Nova Stat Profile Prime Plus and GEM Premier Blood Gas analysers at concentrations within the reference range intervals for all the investigated parameters.

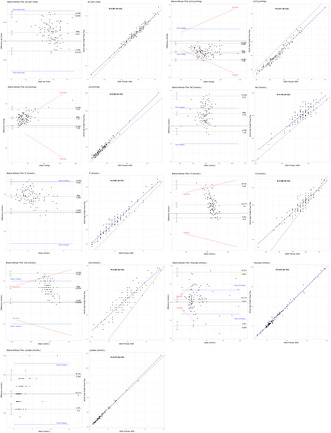

**Summary**

- Current knowledge
  ○ The benefits of point‐of‐care blood gas analysers in emergency and critical care settings are well documented. A paucity of literature is present on the NOVA Stat Profile Prime Plus Analyser, its performance and comparison with other point‐of‐care blood gas systems.
- What this paper contributes to our knowledge
  ○ The verification process of a blood gas analyser is outlined along with the quality performance parameters of the NOVA Stat Profile Prime Plus Analyser. Two blood gas analysers, the NOVA and Gem Premier, were compared and found to have comparable performance. The NOVA Stat Profile Prime Plus Analyser is a reliable device for the analysis of a comprehensive panel of analytes in a POC setting.

## 1 Introduction

Blood gas analysis is a diagnostic procedure that determines the acid–base content and partial pressures of gases in the blood, among other parameters such as glucose and lactate, which are important for the immediate management of patients [1]. Analysis of blood gases at the patient's bedside plays a critical role in both the intensive care and the emergency setting. This is especially so for the monitoring of respiratory, circulatory and metabolic disorders in patients with suspected pathological hypoxaemia, severe metabolic disturbance and/or electrolyte disturbance, critically ill patients and those with acute respiratory failure and toxicology presentation, among others [2]. The clinical use of blood gas analysis has been practised since the 1950s. With advancements in technology, additional parameters were introduced as part of the blood gas analyses test panel, including pH, partial carbon dioxide (pCO_2_), partial oxygen (pO_2_), lactate, glucose and electrolytes including sodium (Na^+^), potassium (K^+^) and chloride (Cl^−^) [3]. These point‐of‐care (POC) analysers now offer accurate results, comparable to the gold standard benchtop blood gas analysers, with results having a much shorter turnaround time [4, 5].

The Nova Stat Profile Prime Plus analyser is one of the recent blood gas analyser models on the market with a comprehensive panel of analytes, including blood gases, electrolytes, metabolites, CO‐Oximetry and 34 calculated parameters from whole blood samples. This analyser uses component cartridge technology for sensors and reagents, with maintenance‐free and nonlysing whole‐blood CO‐Oximetry technology [6]. Further to its original intended use in clinical laboratory settings, its use was further extended to the POC setting [7]. Prior to the introduction of an analyser in a clinical setting, several quality parameters are to be checked to ensure the reliability and performance characteristics of each analyte tested [8]. The Clinical and Laboratory Standards Institute (CLSI) provides guidelines for verification procedures including assessment of accuracy, precision, linearity and comparison [9, 10, 11].

The purpose of this study was to verify the manufacturer's specifications of the Nova Stat Profile Prime Plus Critical Care Analysers (Nova Biomedical), including the within‐ and between‐run precision, accuracy, linearity and bias for several analytes. In addition, a comparison study was made with the GEM Premier 4000 Blood Gas Analyser (Instrumentation Laboratory).

## 2 Materials and Methods

This verification study was conducted at Gozo General Hospital (GGH) Medical Laboratory Services before switching from the GEM Premier 4000 Blood Gas Analyser to the Nova Stat Profile Prime Plus Critical Care Analyser. Parameters analysed in this study were pH, pCO_2_, pO_2_, Na^+^, Cl^−^, K^+^, iCa, lactate and glucose. Data for the precision and bias study were generated using control samples (the Nova Stat Profile Prime Plus Auto‐QC Pack, reference number 57838); linearity was checked using Phoenix Diagnostics Blood Gas/Electrolyte/Metabolite Linearity Control (Reference Number: PH5001) while comparison between the two analysers was done using patient samples submitted during the verification period. Patient samples were tested on the GEM Premier 4000 Blood Gas Analyser, and immediately after, the same sample was run on the Nova Stat Profile Prime Plus Critical Care Analyser. All samples were anonymised before data inputting. All statistical analysis was carried out using R/Rstudio version 2023.03.0 (packages: readxl, MASS, ggpubr, ggplot2, diplyr, outliers, EnvStats, mcr and patchwork). Ethics approval was sought through GGH.

### 2.1 Precision—Guidelines and Materials Used

The precision study was performed according to CLSI EP15‐A3:2014 (File S1; Section 1a) [9], with five repeated measurements of each different control level for each parameter under investigation for 5 days (5 × 5 study) at a frequency of one run per day, yielding a total of 25 results per analyte. The Nova Stat Profile Prime Plus Auto‐QC Pack (Reference Number 57838, Lot 22,045,043) control levels 1, 2 and 3 were used for pH, pCO_2_ and pO_2_, and levels 4 and 5 for Na^+^, K^+^, Cl^−^, ionised calcium (iCa), glucose and lactate. Before each run, it was assured that the internal quality control (IQC) of all levels was in control. During the 5 × 5 study, data were visually inspected for outliers. No runs were flagged as being faulty, and no failed IQC runs were encountered. Analyte measurement against sample number (*N* = 25) was plotted to visually inspect data for any outliers. In this experiment, two types of manufacturer precision claims—repeatability (within‐run imprecision) (σR) and within‐laboratory imprecision (σWL), as reported in the Stat Profile Prime Plus User Manual [6]—were verified. Precision claims were reported as coefficients of variation (CV%) except for pH, where standard deviation (SD) was used.

### 2.2 Bias—Guidelines and Materials Used

The bias study was performed according to CLSI EP15‐A3:2014 (File S1; Section 1b) [8] using statistics from the precision study (*N* = 25 for each control level). In this study, the deviation of the laboratory mean, calculated from the 5 × 5 study, from the assigned target values (mean values) of the Auto‐QC (Stat Profile Plus Blood Gas, CO‐Oximeter and Chemistry Controls Auto‐Cartridge) was determined. The target value was taken as the mean value of the Auto‐QC per parameter per control level (File S1; Section 2).

### 2.3 Linearity—Guidelines and Materials Used

The linearity evaluation study was carried out in compliance with the guidelines provided in CLSI EP06, 2nd Edition (Verification Chapter 4, page 63) (File S1; Section 1c) [11]. This experiment is based on mixtures made from zero to high sample concentrations, where the high sample had a concentration that was higher than the assay's upper limit of quantitation. Each sample of the five‐level linearity set, Phoenix Diagnostics Blood Gas/Electrolyte/Metabolite Linearity Control (Reference Number: PH5001), was analysed twice on the Nova Stat Profile Prime Plus Analyser. Two ampules were used instead of running the analysis twice from the same ampule given that blood gas levels may change when exposed to air. The average value of the replicates was calculated for each sample, the best‐fitted straight line was constructed and the deviation from linearity for each sample was evaluated, considering the acceptable goals for linearity at that concentration. The calculated 95% confidence intervals (CIs) of each level (based on results from this study) were compared to the allowable deviation limit (ADL) (based on Clinical Laboratory Improvement Amendments (CLIA) allowable total error (TE_a_) values) [12]. The former should overlap with the latter for the linearity to be verified as acceptable. Glucose and lactate were not tested for linearity as the Linearity control set used did not give results for glucose and lactate, possibly due to matrix effects.

### 2.4 Comparison Study

The comparison study was performed according to the CLSI EP09c 3rd Edition Guidelines (File S1; Section 1d). Acceptance was based on the CLIA TE_a_ for all analytes except for lactate. For this analyte, the TE_a_ defined by CAP (Collage of American Pathologists) and AAB (American Association of Bioanalysts) was used [9]. Arterial and venous whole blood samples collected in a lithium heparin syringe (BD Vacutainer syringes—3 mL; Reference Number: 364376) submitted to GGH Medical Laboratory Services, between 22nd July and 29th August 2022, were analysed on a validated GEM Premier Blood Gas Analyser (Comparative) and the NOVA Stat Profile Prime Plus Analyser (Candidate) immediately after, within 2 min from each other (total *N* = 103; arterial samples *N* = 35 and venous samples *N* = 68). All samples were submitted to the lab within 15 min following collection. All comparisons were made using the same GEM Premier Blood Gas analyser. Prior to this comparison, the GEM Premier Blood Gas analyser was verified in line with CLIS guidelines. Lots of consumables of the blood gas analysers were not changed during this comparison period. All results for all analytes under investigation were plotted in a box plot to visually assess for outliers. A total of one sample was excluded from the analysis of pCO_2_ and Na^+^, while two samples were excluded from the analysis of K^+^ as they were outliers. Test methodologies for the analytes under study on both the GEM Premier and the NOVA Stat Profile Prime Plus Analyser are found in Table 1. Lab scientists performing testing were trained in the operation of both analysers. Both analysers passed IQC before running patient samples. IQC was scheduled to run automatically at a frequency as recommended by the manufacturer. Both analysers were located next to each other to avoid differences in pCO_2_ and pO_2_ related to exposure to air. The average difference in analysis time between the two analyses (GEM Premier and NOVA) was 2 min. For comparability, the correlation coefficient *R* and average bias (also in percentage) were calculated for each parameter. Bias differences were also visualised on Bland–Altman plots. Regression analysis was also performed, outlining the systemic and proportional differences. The best choice of regression was chosen based on the number of samples, *R* values taken from the correlation analysis, constant SD/CV and presence of outliers (File S1 Section 3). Predictions and bias differences with 95%CIs and/or Bias% were calculated at five medical decision levels (MDLs) which were compared to acceptance criteria. Three MDLs, were concentrations (minimum, maximum and mean) within the reference range [6], while another two MDLs were concentrations specified in the claims reported by the manufacturer [12, 13]. These were the very low and very high concentrations. The predicted values at the specified MDLs were calculated from the regression equation.

**TABLE 1:** Comparison of methodologies employed by the Nova Stat Profile Prime Plus Critical Care Analysers (Nova Biomedical) and the GEM Premier 4000 Blood Gas Analyser (Instrumentation Laboratory).

Parameter (Unit)	Methodology comparison	
	GEM Premier 4000 Blood Gas Analyser^a^	Nova Stat Profile Prime Plus Critical Care Analyser^b^
pH (pH Units)	Direct potentiometry	Direct ISE
pCO_2_ (mmHg)	Direct potentiometry	Severinghaus
pO_2_ (mmHg)	Amperometric	Amperometric
Na^+^ (mmol/L)	Direct potentiometry	Direct ISE
K^+^ (mmol/L)	Direct potentiometry	Direct ISE
Cl^−^ (mmol/L)	Direct potentiometry	Direct ISE
iCa (mmol/L)	Direct potentiometry	Direct ISE
Glucose (mmol/L)	Amperometric	Enzyme/Amperometric
Lactate (mmol/L)	Amperometric	Enzyme/Amperometric

Abbreviation: ISE, ion‐selective electrode.

^a^ Laboratory, I., May 2013. *GEM Premier 4000 Operator's Guide*, USA: Instrumentation Laboratory.

^b^ NOVA, Instructions for Use Manual, Ref 57,819, 2018–2020.

## 3 Results

### 3.1 Precision, Bias and Linearity

**FIGURE 1:**
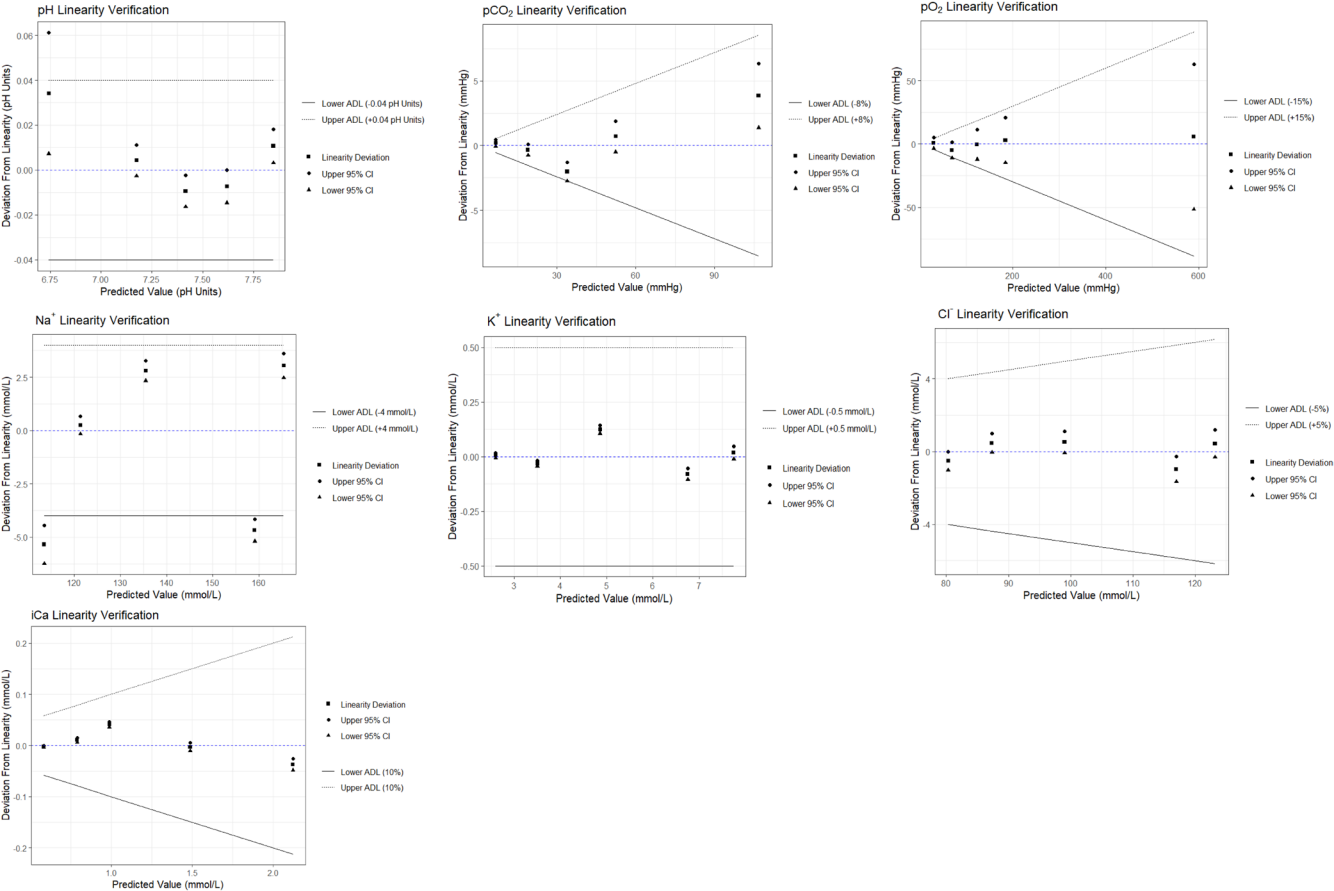
Linearity verification plots for pH, pCO_2_, pO_2_, Na^+^, K^+^, Cl^−^ and iCa. The upper and lower acceptable deviation limits (ADL) are represented by the dotted and solid lines respectively. The upper and lower 95% confidence intervals (CI) are denoted by a circle and triangle, with the square representing the mean.

All parameters under investigation met the manufacturer's within‐run (Table 2) and between‐run (Table 3) precision claims, except for pCO_2_ control level 2 within‐run (CV% 1.5 [manufacturer claim CV% 1.1]) and iCa control level 5 between‐run (CV% 1.42 [manufacturer claim 1.12]). All parameters were found to be within the calculated lower and upper bias limits as indicated in Table 4. The CIs specific for each pool (five pools in total) of the linearity set, for each of the parameters under investigation, all overlapped with the ADL interval (Figure 1), with the exception of Na^+^. The CIs of Levels 2 and 5 (concentrations 169 and 111 mmol/L respectively) of Na^+^ did not overlap with the ADL interval, that is, failed linearity verification.

**TABLE 2:** Within‐run imprecision results of the Nova Stat Profile Prime Plus Analyser using the NOVA Stat Profile Plus Blood Gas, CO‐Oximeter and Chemistry Controls Auto‐Cartridge (Levels 1, 2 and 3 for pH, pCO_2_ and pO_2_; Levels 4 and 5 for Na^+^, K^+^, Cl^−^, iCa, glucose and lactate).

Within‐run imprecision									
	Observed SD (for pH only) and CV% within run (CV_R_%)			Manufacturer's claims (with calculated UVL)					
				Within run SD (for pH only) and pooled CV% (calculated with 20 replicates per run on 3 analysers)			Within run SD (for pH only) and CV% (calculated using controls in duplicates over 20 days, 2 runs per day)		
	Level 1	Level 2	Level 3	Level 1 (UVL)	Level 2 (UVL)	Level 3 (UVL)	Level 1 (UVL)	Level 2 (UVL)	Level 3 (UVL)
pH	0.001	0.001	0.002	0.002 (0.003)	0.001 (0.001)	0.003 (0.004)	0.003 (0.004)	0.001 (0.001)	0.001 (0.001)
pCO2	1.2	1.5	0.55	2.3 (3.1)	0.8 (1.1)	4.7 (6.3)	1.9 (2.5)	0.8 (1.1)	1.6 (2.1)
pO2	1.12	0.63	0.38	2.0 (2.68)	1.7 (2.28)	1.3 (1.74)	5.0 (6.7)	3.0 (4.02)	2.3 (3.08)
	Level 4	Level 5		Level 4 (UVL)	Level 5 (UVL)		Level 4 (UVL)	Level 5 (UVL)	
Na^+^	0.07	0.07		0.1 (0.13)	0.2 (0.27)		0.3 (0.40)	0.4 (0.52)	
K^+^	0.05	0.12		0.1 (0.13)	0.7 (0.94)		0.5 (0.67)	0.8 (1.07)	
Cl^−^	0.14	0.13		0.2 (0.26)	0.2 (0.27)		0.9 (1.21)	1.2 (1.61)	
iCa	0.37	0.24		0.3 (0.40)	0.7 (0.92)		1.0 (1.34)	0.7 (0.92)	
Glu	0.57	0.48		0.8 (1.07)	1.1 (1.47)		1.5 (2.01)	1.7 (2.28)	
Lac	0	0.44		0 (0)	0.4 (0.54)		2.5 (3.35)	1.9 (2.55)	

Abbreviations: CV, coefficient of variation; Glu, glucose; Lac, lactate; SD, Standard deviation and UVL, upper verification limit.

**TABLE 3:** Within‐lab imprecision results of the Nova Stat Profile Prime Plus Analyser using the NOVA Stat Profile Plus Blood Gas, CO‐Oximeter and Chemistry Controls Auto‐Cartridge (Levels 1, 2 and 3 for pH, pCO_2_ and pO_2_; Levels 4 and 5 for Na, K, Cl, iCa, glucose and lactate).

Imprecision within‐lab (between runs)						
	Observed SD (for pH only) and CV% within run (CV_R_%)			Manufacturer's claims with calculated UVL		
				Total SD (for pH only) CV% within lab (calculated using controls in duplicates over 20 days, 2 runs per day)		
	Level 1	Level 2	Level 3	Level 1 (UVL)	Level 2 (UVL)	Level 3 (UVL)
pH	0.004	0.002	0.002	0.005 (0.008)	0.011 (0.02)	0.005 (0.008)
pCO2	3.7	1.89	3.18	4.7 (7.8)	3.4 (5.6)	4.1 (6.8)
pO2	1.15	0.70	0.79	4.7 (7.8)	2.8 (4.6)	2.4 (3.98)
	Level 4	Level 5		Level 4 (UVL)	Level 5 (UVL)	
Na^+^	0.08	0.19		0.2 (0.31)	0.3 (0.47)	
K^+^	0.05	0.17		0.2 (0.26)	0.6 (0.81)	
Cl^−^	0.18	0.14		0.9 (1.29)	1.2 (1.66)	
iCa	0.37	1.42		1.0 (1.33)	0.7 (1.12)	
Glu	1.25	0.71		2.0 (2.70)	1.9 (2.95)	
Lac	0	0.64		3.5 (4.48)	1.3 (2.08)	

Abbreviations: CV, coefficient of variation; Glu, glucose; Lac, lactate; SD, Standard deviation and UVL, upper verification limit.

**TABLE 4:** Bias (%) results of the Nova Stat Profile Prime Plus Analyser using the NOVA Stat Profile Plus Blood Gas, CO‐Oximeter and Chemistry Controls Auto‐Cartridge (Levels 1, 2 and 3 for pH, pCO_2_ and pO_2_; Levels 4 and 5 for Na^+^, K^+^, Cl^−^, iCa, glucose and lactate).

Calculated Bias and Bias%						
	Level	Target Value	Obs. Mean	Calc. LL	Calc. UL	Bias (%)
pH	1	7.243	7.245	7.214	7.272	−0.03
	2	7.432	7.438	7.407	7.465	−0.08
	3	7.625	7.639	7.607	7.665	−0.18
pCO_2_ (mmHg)	1	54.9	56.5	46.2	60.4	−2.91
	2	40.25	38.8	34.3	44.1	3.60
	3	21.95	19.3	13.0	21.0	12.07
pO_2_ (mmHg)	1	61.65	64.8	53.0	72.6	−5.11
	2	107.75	108.2	99.1	118.7	−0.42
	3	148.05	143.8	129.4	158.8	2.87
Na^+^ (mmol/L)	4	141.4	143.2	137.1	144.9	−1.27
	5	115.8	117.1	111.9	119.7	−1.12
K^+^ (mmol/L)	4	3.94	4.01	3.68	4.18	−1.78
	5	6.22	6.36	5.93	6.51	−2.25
Cl^−^ (mmol/L)	4	126.85	127.7	121.8	130.6	−0.67
	5	98.25	98.9	93.8	102.6	−0.66
iCa (mmol/L)	4	1.075	1.08	1.00	1.16	−0.47
	5	1.48	1.45	1.36	1.60	2.03
Glucose (mg/dL)	4	80.5	82	73	89	−1.86
	5	273.5	287	252	301	−4.94
Lactate (mmol/L)	4	2.0	2.0	1.7	2.3	0.00
	5	6.95	7.1	6.2	7.6	−2.16

Abbreviations: Calc. LL, calculated lower limit; Calc. UL, calculated upper limit and Obs. Mean, Observed mean.

### 3.2 Comparability

**FIGURE 2:**
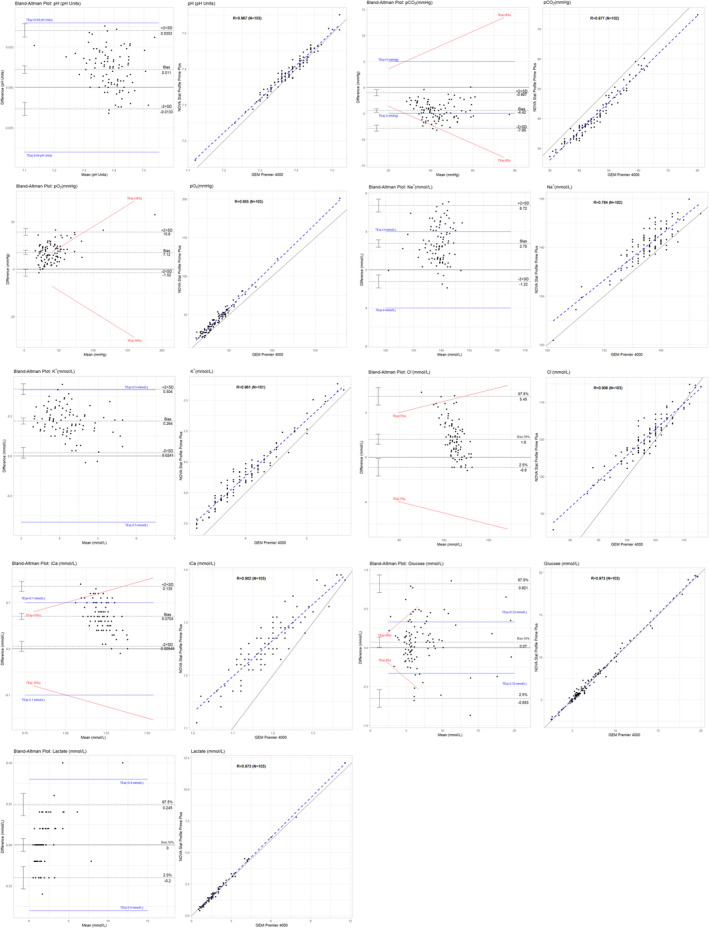
Bland–Altman plots and scatter plots of the comparative study between the Nova Stat Profile Prime Plus and the Gem Premier 4000 analyser.

The correlation coefficient (R) of pH, pCO_2_, pO_2_, K^+^, glucose and lactate was all above 0.95, ranging from 0.96 to 0.98 (File S1; Section 4). Cl^−^, iCa and Na^+^ had an *R* value of 0.90, 0.91 and 0.78, respectively. Regression slopes of all the parameters ranged from 0.72 to 1.10, and the intercept ranged from −4.22 to 30.88. No proportional and/or systematic bias was observed for Na^+^, glucose and lactate. pCO_2_ showed systematic bias, while pO_2_ had proportional bias. Cl^−^, K^+^ and iCa had both proportional and systematic bias (Table 5). This bias is also visible in the Bland–Altman and scatter plots (Figure 2). The predicted values at different MDL and the MDL Bias with the 95% CI were calculated (Table 5). Any 95% CI not falling within the TE_a_ range was deemed unacceptable. The MDL bias% was also calculated to compare with the TE_a_ that is given in percentage. Where both unit and percentage TE_a_ are given, the one that is largest was compared to the respective TE_a_. Na^+^, Cl^−^, and iCa were the only parameters that had a 95% CI range or Bias% larger than the TE_a_ at particular MDLs. However, the MDLs that were out of range were not part of the reference range interval.

**TABLE 5:** Predicted results of all the parameters at different medical decision limits.

Parameter	Regression Type (Equation)	Sys. Diff. (95% CI) Intercept	Prop. Diff. (95% CI) *Slope*	MDL^a^	Pred.	MDL Bias (95% CI)	MDL Bias %	Accept. Criteria (TE_a_)
pH	Deming (*y* = 0.36 + 0.95*x*)	0.35 (0.06, 0.65)	0.95 (0.91, 0.99)	7.35 7.40 7.45 7.10 7.60	7.36 7.41 7.46 7.13 7.60	−0.01(−0.02, −0.01) −0.01(−0.02, − 0.01) −0.01(−0.01, 0.00) −0.02(−0.04, − 0.01) −0.00(−0.01, 0.01)	−0.17 −0.14 −0.10 −0.34 0.00	±0.04 pH units
pCO_2_ (mmHg)	Ordinary Least Squares (*y* = −3.12 + 0.97 *x*)	−3.12 (−4.95, −1.29)	0.97 (0.93, 1.01)	35 40 45 20 75	30.88 35.73 40.59 16.31 69.73	4.12(3.59, 4.66) 4.27(3.87, 4.67) 4.41(4.07, 4.74) 3.69(2.63, 4.76) 5.27(4.05, 6.48)	11.77 10.67 9.80 18.47 7.02	± 5 mmHg or 8%
pO_2_ (mmHg)	Passing–Bablok (*y* = 2.94 + 1.10*x*)	2.94 (0.93, 5.15)	1.10 (1.05, 1.15)	83 95.5 108 40 160	94.54 108.33 122.13 47.08 179.52	−11.54(−15.10, − 7.98) −12.83(−17.21–8.45) −14.13(−19.35, − 8.90) −7.08(−8.57, − 5.60) −19.52(−28.43, − 10.68)	−13.90 −13.44 −13.08 −17.71 −12.20	±18%
Na^+^ (mmol/L)	Deming (*y* = −4.22 + 1.05*x*)	−4.22 (−20.29, 11.84)	1.05 (0.93, 1.17)	136 140 146 120 160	138.67 142.88 149.18 121.86 163.89	−2.6(−3.09, − 2.26) −2.88(−3.33, − 2.42) −3.18(−4.09, − 2.27) −1.86(−3.61, − 0.12) −3.89(−6.10, − 1.68)	−1.97 −2.05 −2.18 −1.55 −2.43	±4 mmol/L
K^+^ (mmol/L)	Deming (*y* = 0.54 + 0.93*x*)	0.54 (0.38, 0.70)	0.93 (0.89, 0.97)	3.5 4.3 5.1 2.8 6	3.80 4.55 5.29 3.15 6.13	−0.30(−0.33, −0.27) −0.25(−0.27, −0.22) −0.19(−0.24, −0.14) −0.35(−0.40, −0.29) −0.13(−0.21, −0.04)	−8.60 −5.74 −3.77 −12.45 −2.18	±0.5 mmol/L
Cl^−^ (mmol/L)	Deming (*y* = 30.88+ 0.72*x*)	30.88 (26.05, 35.71)	0.72 (0.67, 0.77)	98 102 106 79.3 128.5	101.33 104.21 107.08 87.89 123.26	−3.33(−3.70, − 2.97) −2.22(−2.45, − 1.97) −1.08(−1.34, − 0.83) −8.59(−9.86, − 7.32) 5.24(3.93, 6.55)	−3.40 −2.17 −1.02 −10.83 4.08	±5%
iCa (mmol/L)	Deming (*y* = 0.36 + 0.76*x*)	0.37 (0.28, 0.43)	0.76 (0.69, 0.83)	1.09 1.195 1.3 0.8 1.54	1.18 1.26 1.34 0.96 1.53	−0.09(−0.010, −0.09) −0.07(0.07, −0.06) −0.04(−0.05, −0.03) −0.16(−0.19, −0.14) 0.01(−0.01, 0.04)	−8.58 −5.71 −3.31 −20.42 0.96	±0.10 mmol/L or 10%
Glucose (mmol/L)	Passing Bablok (*y* = −0.021 + 1.012*x*)	−0.02 (−0.21, 0.15)	1.01 (0.99, 1.04)	3.61 4.44 5.27 2.22 6.66	3.63 4.47 5.31 2.23 6.72	−0.02(−0.12, 0.08) −0.03(−0.12, 0.06) −0.04(−0.12, 0.04) −0.01(0.13, 0.12) −0.06(−0.13, 0.01)	−0.63 −0.74 −0.82 −0.26 −0.90	±0.33 mmol/L or 8%
Lactate (mmol/L)	Passing Bablok (*y* = −0.07 + 1.06*x*)	−0.07 (0.13, 0.00)	1.06 (1.00, 1.09)	0.70 1.60 2.50 2.00 6.00	0.67 1.62 2.57 2.04 6.26	0.03(0.00, 0.06) −0.02(−0.04, 0.01) −0.07(−0.09, − 0.04) −0.04(−0.06, − 0.02) 0.26(−0.03, 0.19)	4.757 −1.046 −2.671 −1.949 −4.356	±0.40 mmol/L

Abbreviations: accept, acceptance; MDL, medical decision limit; Pred, predictive value and TE, allowable total error.

^a^ The first three MDLs are concentrations (minimum, maximum and mean) within the reference range, while the 4th and 5th MDLs are concentrations within the claims reported by the manufacturer (NOVA, Instructions for Use Manual 2018–2020).

## 4 Discussion

POC devices include both portable benchtop and handheld analysers [14]. POC blood gas analysers provide rapid blood gas, electrolytes, glucose and lactate analysis, which is important in the evaluation of acid–base and oxygenation status, supplemental oxygen therapy effectiveness, sepsis treatment and patient circulation and metabolic progress [5]. This is an asset not only in the treatment and monitoring of critically ill patients but also in emergency settings. This was very apparent during the COVID‐19 pandemic but is still of great support to medical staff for immediate patient management, with the increasing incidence of respiratory challenges [15, 16]. The preanalytical phase poses many risks of failures and/or erroneous blood gas results owing to the instability of blood gases in blood, which can potentially impact patient care [17]. Thus, having a POC analyser to provide timely and reliable results of a panel of analytes on which medical decisions can be taken is critical. With the advancement in technology, many more POC devices are being released on the market; however, the verification process of these devices should be the same as that of other laboratory analysers, including the application of a quality control system.

While many blood gas analysers are now available, the results vary widely due to analyser methodological differences, including different calibration procedures. Therefore, a verification process prior to the introduction of an analyser in the diagnostic setting is important, where imprecision, inaccuracy and linearity are verified, and comparison experiments are done between comparative and candidate methods [18]. While verification of blood gas analysers poses unique challenges due to sample instability, especially for pCO_2_ and pO_2_ parameters, this study gives a detailed step‐by‐step guide on the verification experimental process in line with CLSI guidelines to fulfil ISO accreditation requirements.

Overall, the NOVA Stat Profile Prime Plus Analyser performed well in the precision and bias studies. This is in line with findings reported by others, where precision CV% results for analytes tested on the NOVA analyser were within the manufacturer's specifications [19]. Linearity across the interval tested was verified for all parameters except for Na^+^, which showed borderline performance, as linearity was not verified across all the concentrations tested. This warrants further investigation as analyser/sensor‐specific issues and/or matrix effects of the linearity set cannot be excluded.

The NOVA analyser compared well with the GEM Premier Analyser, with only Na^+^, Cl^−^, and iCa exceeding their respective TE_a_. While it is known that differences in Na^+^ results can be attributed to high blood lipid and protein levels when using indirect potentiometry [20], this cannot explain the observations reported here, as both the NOVA and GEM Premier analysers use a direct potentiometry test methodology. This lack of correlation was not reported by others, possibly due to the fact that it was only observed at MDLs that were out of the reference range interval, thus having minimal clinical implications. In addition, comparison studies between the NOVA and the GEM Premier analysers are limited. The Nova analyser compared well with the Radiometer ABL800 FLEX/Abbott i‐STAT Chem8+ POCT analysers [19] and showed a good agreement with a central laboratory analyser for Na^+^ and K^+^ [21]. The lactate estimation by the Nova Stat Profile Prime Plus Analyser was also found to significantly predict any underlying disorders and poor outcomes [21].

Although samples were immediately tested upon submission to the lab as per manufacturers’ recommendations, and minimal delays were present between testing on comparative and candidate analysers, the possibility that air exposure altered blood gas levels cannot be excluded. The preanalytical phase, including sample collection, patient preparation and transportation, was not accounted for in this study. Comparison was done with patient samples, and the extremely high and low ranges were not available for all analytes under investigation. These results were observed using the CLIA TEa values. The use of other TEa values may lead to different conclusions. Conclusions on the performance of the analyser at concentrations lower and/or higher than the stated levels included in this study cannot be drawn.

## 5 Conclusion

In conclusion, the Nova Stat Profile Prime Plus Analyser meets the manufacturer's precision and bias claims. Linearity was confirmed for pCO_2_, pO_2_, K^+^, Cl^−^ and iCa. A good correlation was observed between the Nova Stat Profile Prime Plus and GEM Premier Blood Gas Analysers at concentrations within the reference range intervals for all the investigated parameters. Based on the results from the verification process, it can be concluded that the Nova Stat Profile Prime Plus Analyser meets the claimed quality performance parameters and is a reliable device for the analysis of a comprehensive panel of analytes in a POC setting.

## Author Contributions

J.C. has done the literature search. Data collection was done by R.A. and other scientific staff at GGH Medical Laboratory Services. J.C. and R.A. were involved in the study design, and data analysis was done by J.C. J.C. prepared the manuscript and R.A. reviewed it. The study was conducted at Gozo General Hospital, Victoria, Gozo, Malta.

## Conflicts of Interest

The authors declare no conflicts of interest. The funders had no role in the design of the study; in the collection, analyses or interpretation of data; in the writing of the manuscript; or in the decision to publish the results.

## Supporting information


Data S1.

## Data Availability

The data that support the findings of this study are available from the corresponding author upon reasonable request.
