# Supplementary material for: The Verification Process of a POC Blood Gas Analyser—The Nova Stat Profile Primer Plus Analyser
Source: J Clin Lab Anal. 2025 Mar 25;39(7):e70006. doi: 10.1002/jcla.70006 (PMC11981951; doi:10.1002/jcla.70006)
Supplement: Supplementary file 1 — Data S1. [file JCLA-39-e70006-s001.docx]

**Supplementary Material:**

**Section 1a. Statistical analysis for precision study**

The mean (*x̄*) of the 25 observed control values was calculated for each control level using the formula,

x̄ = Σx/n

where *x* is the observed control value and *n* is the number of observations. The standard deviation (SD) was calculated using the formula,

s = sqrt [ s^2] = sqrt [ Σ (x_i_- x̄)^2 / ( n - 1 ) ]

Analyte measurement against sample number (N=25) was plotted to visually inspect data for any outliers. Grubbs Test was also used to detect outliers. A single result per control level, at most, was considered a statistical outlier. Two results, at most, were regarded as statistical outliers for the entire study's samples. A result was considered an outlier if it deviated more than G standard deviations from the sample mean. The sample mean and standard deviation were derived from all 25 results in the sample, including the suspected outlier. The Grubbs’ factor G depends on the number of samples *N* and it is obtained from Table 3 of the CLSI EP15-A3:2014 guideline [8]. In this case, since N=25, Grubbs was 3.135. Grubbs limits [lower limit (LL) and higher limit (HL)] were calculated using the formula:

mean ±G x SD

The One-Way Analysis of Variance (ANOVA) was used to calculate within-group and between group variances. This was the basis for the calculation of repeatability (within-run variance) and within-laboratory imprecision (between run variance) estimates for each control level. In Table S1 the SS (sum of squares) and DF (Degrees of freedom) are given. The MS (mean squares) is calculated by dividing the SS with DF. For between run these are denoted as SS1, DF1 and MS1. For within-run these are denoted as SS2, DF2 and MS2. SS total is the variance estimation of the whole sample. DF total is the degrees of freedom of the whole sample (count -1) which in this case it was 24 (25-1).

**Table S1** One-way ANOVA descriptive table.

| **Source of Variation** | **SS** | **DF** | **MS** |
| --- | --- | --- | --- |
| Between-run | SS1 | DF1 | MS1 |
| Within-run | SS2 | DF2 | MS2 |
| Total | SS total | DF total |  |

SS=Sum of Squares, DF=Degrees of Freedom, MS=Mean Squares

The repeatability variance (V_w_) was the MS2 and the between run variance (V_B_) was calculated as MS1-MS2/runs:

V_w_=MS2

V_B_=MS1-MS2

To compare with manufacturer claims, the variance was either converted to SD by square root or CV (calculated from the SD). The SD repeatability (S_R_), SD between runs (S_B_) and SD within laboratory (total precision) (S_WL_) were calculated using the variance as follows:

S_R_ = √V_W_

S_B_ = √V_B_

S_WL_ = √(V_W_)+V_B_

The coefficient of variation (CV%) was calculated using the formula:

CV=mean/SD×100

The CV repeatability (CV_R_ %), the CV between runs (CV_B_ %) and CV within lab (total precision) (CV_WL_ %) where calculated as follows:

CV_R_= S_R_ × 100/x

CV_B_= S_B_ × 100/x

CV_WL_= S_WL_ × 100/x

where *x* is the grand mean of all results for the sample.

If the actual imprecision is, on average, equal to the claimed imprecision, then 50% of the time the observed imprecision will be greater than the reported imprecision owing to chance alone. An upper verification limit (UVL) was determined for each claim in order to restrict the number of failures to verify the manufacturer's claims that can be attributed to random chance. When the claim is true, the UVL indicates the upper 95^th^ percentile expected for imprecision estimates derived from an experiment with a size and design comparable to the user's precision verification study. This safeguards the user from failing unfairly because of chance alone (more than 5 percent of the time) by making the imprecision verification acceptance criterion the UVL rather than the claim itself. The UVL was calculated from the degrees of freedom (df). For repeatability (UVL_W_), the degrees of freedom (df) was calculated using the formula:

df_R_ = N - k

where *N* is total number of results and *k* is number of runs. For the within-lab imprecision (UVLWL), first the claims ratio (ρ) was calculated as follows:

ρ =σ_WL_/σ_R_ =(%CV_WL_)/(%CV_R_ )

The df_WL_ were determined from Table 6 of the CLSI EP15-A3:2014 guidelines [8]. In this case for 5 runs at *ρ* of 1.43, the df_WL_ was 10. Using the df = df_WL_, the UVL factor *F* was determined from Table 7 of the CLSI EP15-A3:2014 guidelines [8]. The UVL was calculated by multiplying factor *F* with the relevant claim of the manufacturer either as SD or CV%:

UVL = F × SD or

UVL = F × %CV

For UVLW of 20 df from Table 6 the F value from Table 7 was 1.34 when having a total of 3 control samples (in the case of 2 control samples it was 1.31). For UVL_WL_ the *F* value of 10 df and a total of 3 control samples was 1.47. For pH claim was in SD while for all the other parameters both SD and CV% were provided by the manufacturer.

**Section 1b. Statistical analysis for bias study**

From the precision study the SW_L_ % and S_R_ % were used to calculate the standard error of the mean (SE(x)) using the formula,

SE(x̄) = √ (1/nRun [s_WL_^2^ – ((nRep-1/nRep)s_R_^2^))])

where nRun is the number of runs (5 in our case); nRep = 5. S_WL_ and S_R_ were obtained from the precision study. The SD of the reference material was used to calculate the uncertainty from which the standard error of the reference material (target value) (se_RM_) was calculated, using the formula below. Uncertainty is expressed in the form of a standard deviation called standard uncertainty u(x̄), or in an expanded form U (U= +/- k × u).

se_RM_ = U / k

where U is the expanded uncertainty and k is the uncertainty of SD which is 2.0 at 95% confidence interval (CI). The combined standard error of mean and target value (se_c_) was calculated using the formula,

se_c_ = √ se_x̄_^2^ + se_RM_^2^

where the se_RM_ was the standard error of the reference material. A verification interval was calculated from the degrees of freedom after calculating the combined standard errors and *m* value. The degrees of freedom (df) was calculated as,

df_x̄_ = nRun - 1

For this study the df was 4 (5 – 1). The combined degrees of freedom (df_c_) was calculated using the formula,

df_c_ = df_x̄_ × (se_c_/se_x̄_)^4^

The multiplier *m* value (*m* was set to the Student’s quantile for a probability of 0.975) was calculated as,

m = t_1-α / 2×_*_nSam, v_*

where *nSam* is the number of samples (for 1 sample at 95% confidence interval significant level is 0.975), alpha level is set at 0.05. *t* is derived from the t-value table. The Verification interval (VI) was calculated using the formula:

VI = TV ± (m × se_c_)

This provides a range TV- and TV+ in which the lab mean must fall to pass the study i.e. meeting the manufacturer’s claim. The percentage bias (bias %) was calculated using the formula:

Bias (%) = (Target Value-Observed Value)/(Target Value)×100

**Section 1c. Statistical analysis for linearity verification**

The assigned target levels of the linearity set (Phoenix Diagnostics Blood Gas / Electrolyte / Metabolite Linearity Control; Reference number: PH5001; Lot number: 00851) used in this study were given by the manufacturer (Refer to Table S2).

**Table S2.** Phoenix Diagnostics Blood Gas / Electrolyte / Metabolite Linearity Control range and mean of each level.

| **Parameter** | **Unit** | **Level 1**  **(min- x̄ - max)** | **Level 2**  **(min- x̄ - max)** | **Level 3**  **(min- x̄ - max)** | **Level 4**  **(min- x̄ - max)** | **Level 5**  **(min- x̄ - max)** |
| --- | --- | --- | --- | --- | --- | --- |
| pH | pH units | 6.83-6.88-6.93 | 7.17-7.22-7.27 | 7.36-7.41-7.46 | 7.52-7.57-7.62 | 7.70-7.75-7.80 |
| pCO_2_ | mmHg | 113-125-137 | 55-63-71 | 38-42-46 | 21-25-29 | 7-11-15 |
| pO_2_ | mmHg | 9-24-39 | 48-63-78 | 104-116-128 | 164-176-188 | 485-575-665 |
| Na^+^ | mmol/L | 106-111-116 | 116-121-126 | 134-139-144 | 155-160-165 | 172-177-182 |
| K^+^ | mmol/L | 1.9-2.4-2.9 | 2.9-3.4-3.9 | 4.4-4.9-5.4 | 6.5-7.0-6.5 | 7.6-8.1-10.6 |
| Cl^-^ | mmol/L | 72-77-82 | 81-56-91 | 96-101-106 | 119-124-129 | 127-132-137 |
| iCa | mmol/L | 2.07-2.57-3.07 | 1.53-1.68-1.83 | 0.88-0.98-1.08 | 0.60-0.70-0.80 | 0.31-0.41-0.51 |

Min=minimum, x̄=mean, max=maximum

Each level was assayed twice and the mean of each level was calculated suing the formula:

x̄ = Σx/n

The standard deviation of the two readings was calculated using the formula,

s = sqrt [ s^2 ] = sqrt [ Σ (x_i_- x̄)^2 / ( n - 1 ) ]

A plot of measured values (duplicates of replicate 1 and replicate 2) for each of the 5 concentration levels (linearity set) for each parameter was plotted against the proportion of high pool. This plot was used to inspect the linearity integrity of the data. The sigma value was calculated for each level and used to calculate the weights for the weighted regression. A precision profile plot was generated by plotting the SD against the mean of each concentration levels, from an unweighted regression and forced 0 intercept (straight line fitted to the data). The model from the precision profile (SD = slope × mean) was used to estimate sigma values for the first four pools while the actual SD was used for the low pool (pool 5). The proportions of the levels were defined with the highest concentration (pool 1) taken as 1 as follows:

Proportion = (Target Value)/(High Pool)

Based on precision profile and SD of the Low pool, the weights were calculated as follows:

weight = 1/sigma value2

Unweighted regression was performed. The weights for pool 5 was calculated from the actual SD. The predicted value was determined from the weighted regression (linear model plot). The deviation from linearity, that is the deviation of the observed mean (mean of the two readings/replicates) and the predicted value was calculated as follows,

deviation = observed value mean – predicted value.

The 95% CI of deviation from linearity was based on the sigma values used in the weighting. The lower and upper confidence limits around the deviation of the mean of *R* replicates (i.e. the measured values) from the fitted straight line (i.e. the predicted value) at the specific concentration levels were determined as follows:

[L_i_, U_i_] = (ȳ - ŷ_i_) ± Z_1 – α/2_(σ_i_/√R)

where L_i_ = Lower limit of CI, U_i_ = Upper limit of CI, ȳ = mean of replicates, ŷ_i_ = concentration predicted from fitted straight line (predicted value), σ_i_ = sigma value or SD value in case of the low pool; α=1-(1-αn)1/n; where: αn = 0.05 (95%); n=5. Adjusted to produce CI limits around individual means. Z_1 – α/2_ = (1-α/2)-quantile of the normal distribution (value was looked up in z table, for example 0.9949 = 2.57); R= number of replicates. The lower limit confidence interval (LLCI) and upper limit confidence interval (ULCI) were calculated as follows,

Lower Limit = Deviation – CI

Upper Limit = Deviation + CI

The acceptable deviation limit (ADL) used the TE_a_ as defined by CLIA either in percentage (%) or the unit of the parameter. In the case of the unit, the same value is used for all levels while in the case of % the following calculation was used:

ADL = %TE_a_ ×Predicted / 100.

Plots of deviation from linearity against predicted values for each concentration (each level of the linearity set) for each parameter under investigation were constructed. The upper limit of CI and lower limit of CI were also plotted. The CIs specific to each pool (5 pools in total) were compared with the ADL. Linearity across the interval in question was verified only when all CIs overlapped with the ADL intervals.

**Section 1d. Statistical analysis for comparison study**

A scatter plot was constructed for each parameter under investigation with the results from the comparative method (GEM® Premier) plotted on the x axis and results from the candidate method (NOVA) plotted on the y axis. Descriptive statistics including sample size, range (lowest and highest value), mean and 95%CI, median and 95%CI, variance and SD, %CV and the standard error of the mean were determined. The Anderson Darling test was used to test for normality. A p-value cut-off of 0.05 was used. Grubbs (double sided) was used to test for outliers at alpha-level 0.05. Rosner’s test was used for the detection of more than one outlier when Grubbs test was significant (<0.05) for one outlier. Correlations were done using Pearson’s correlation for normally distributed data or Spearman’s for non-normal data. A p-value cut-off of 0.05 was used. For non-normal data the median was used while for normally distributed data the mean was used. The differences between the comparative and candidate method were calculated and plotted (difference plot) to check for constant SD or constant CV. Average bias and the 95% CI and the % Bias were calculated from the Bland Altman plots. The Bland Altman plot mean %bias was calculated as follows,

%bias = ([comp – cand] / mean of comp & cand * [100].

Confidence standard error (se) (the standard error to calculate the confidence interval) was calculated using the critical value of t. This was obtained from the two-tailed student t-distribution, based on the alpha (0.05) and n (sample number) as follows:

ŷ ± t_crit_ × se

where, t_crit_ is the critical value of the t distribution for the specified significance level α divided by 2. The *se* was calculated as follows:

se = s_yx_ √(1/n) + ((x -x̄)^2^/SS_x_)

where the s_yx_ is the standard estimate of the error which returns the standard error of the predicted y-value for each x in the regression. The standard error is a measure of the amount of error in the prediction of y for an individual x, n = sample number, x is the MDL and the x̄ is the mean of the comparative method; SS_x_ calculates the sum of the squares of the values in a given range. The lower 95% CI was calculated by subtracting the predicted value and then multiplied with the confidence standard error. The upper 95% CI was calculated by the addition of the predicted value and then multiplied with the confidence standard error. Bias was calculated by subtracting the MDL from the predicted value. The lower 95% CI Bias was calculated by subtracting the upper 95% CI from the MDL. The upper 95% was calculated by subtracting the lower 95% CI from the MDL. The %Bias was calculated as follows:

MDL-Predicted/MDL x 100

Acceptance was based on the CLIA TE_a_ obtained from Westgard (2023) and García-Fernández et al. (2022). [12, 14]. TE_a_ values for lactate were taken from CAP (Collage of American Pathologists) and AAB (American Association of Bioanalysts).

**Section 2.** Mean (x̄) and ranges (min – max) of each parameter at different control levels.

| **Parameter** | **Unit** | **Control 1**  (min- x̄ - max) | **Control 2**  (min- x̄ - max) | **Control 3**  (min- x̄ - max) | **Control 4**  (min- x̄ - max) | **Control 5**  (min- x̄ - max) |
| --- | --- | --- | --- | --- | --- | --- |
| pH | pH units | 7.213-7.243-7.273 | 7.406-7.436-7.466 | 7.606-7.636-7.666 |  |  |
| pCO_2_ | mmHg | 46.3-53.3-60.3- | 34.2-39.2-44.2 | 17-21.0-25.0 |  |  |
| pO_2_ | mmHg | 52.8-62.8-72.8 | 98.9-108.9-118.9 | 129-144.1-159.1 |  |  |
| Na^+^ | mmol/L |  |  |  | 137.0-141.0-145.0 | 111.8-115.8-119.8 |
| K^+^ | mmol/L |  |  |  | 3.68-3.93-4.18 | 5.92-6.22-6.52 |
| Cl^-^ | mmol/L |  |  |  | 121.7-126.2-130.7 | 93.7-98.2-102.7 |
| iCa | mmol/L |  |  |  | 1.00-1.08-1.16 | 1.36-1.48-1.60 |
| iMg | mmol/L |  |  |  | 0.57-0.64-0.71 | 1.06-1.21-1.36 |
| Glucose | mg/dL  mmol/L |  |  |  | 73-81-89  4.1-4.5-4.9 | 252-277-302  14.0-15.4-16.8 |
| Lactate | mmol/L |  |  |  | 1.7-2.0-2.3 | 6.2-6.9-7.6 |

**Section 3**. Parameters used for the selection of the best method for the regression analysis as published by Westgard (2020).

| **Number of samples** | **R** | **Constant SD or CV** | **Outliers?** | **Best Choice** |
| --- | --- | --- | --- | --- |
| Less than 20 | Any | Either | N/A | Difference plot |
| 20 or more | >0.975 | SD | None | Least Squares |
| 20 or more | >0.975 | CV | None | Weighted Least Squares |
| 20 or more | <0.975 | SD | None | Deming |
| 20 or more | <0.975 | CV | None | Weighted Deming |
| More than 40 at very least | Any | Either | None or some | Passing-Bablok |

**Section 4**. Comparison results for each evaluated parameter between the GEM® Premier 4000 and NOVA Stat Profile Prime Plus® analysers.

| **Parameter** | **N**^†^ | **R** | **Av. Bias** | **Av. Bias %** |
| --- | --- | --- | --- | --- |
| **pH** | 103 | 0.967* | 0.01 (Mean)  0.01 (SD) | 0.15 |
| **pCO_2_ (mmHg)** | 102 | 0.977 | -4.42 (Mean)  1.71 (SD) | -9.90 |
| **pO_2_ (mmHg)** | 103 | 0.955* | 7.12 (Mean)  4.32 (SD) | 22.00 |
| **Na^+^ (mmol/L)** | 102 | 0.784 | 2.75 (Mean)  1.99 (SD) | 2.00 |
| **K^+^ (mmol/L)** | 101 | 0.961* | 0.26 (Mean)  0.12 (SD) | 6.78 |
| **Cl^-^ (mmol/L)** | 103 | 0.906 | 1.6 (Median)  2.3 (IQR) | 1.75 |
| **iCa (mmol/L)** | 103 | 0.902 | 0.07 (Mean)  0.03 (SD) | 6.07 |
| **Glucose (mmol/L)** | 103 | 0.973* | 0.07 (Median)  0.34 (IQR) | 1.26 |
| **Lactate (mmol/L)** | 103 | 0.973* | 0.00 (Median)  0.2 (IQR) | -1.18 |

N=Sample number, R=Correlation Coefficient, Av. Bias=Average Bias, Sys. Diff=Systematic Difference, Prop. Diff=Proportional Difference, CI=Confidence Interval, SD=Standard Deviation, IQR=Inter Quartile Range.

*spearman’s correlation coefficient was calculated

^†^1 sample was excluded from the analysis of pCO2 and Na while 2 samples were excluded from the analysis of K as they were outliers.
